# Ferroelectrics under the Synchrotron Light: A Review

**DOI:** 10.3390/ma9010014

**Published:** 2015-12-30

**Authors:** Luis E. Fuentes-Cobas, María E. Montero-Cabrera, Lorena Pardo, Luis Fuentes-Montero

**Affiliations:** 1 Centro de Investigación en Materiales Avanzados, Miguel de Cervantes 120, Compejo Industrial Chihuahua, Chihuahua 31136, Mexico; elena.montero@cimav.edu.mx; 2 Instituto de Ciencia de Materiales de Madrid, Consejo Superior de Investigaciones Científicas, Cantoblanco, Madrid E-28049, Spain; lpardo@icmm.csic.es; 3 Diamond Light Source Ltd., Beamline I24, Diamond House, Harwell Science and Innovation Campus, Didcot, Oxfordshire OX11 0DE, UK; luis.fuentes-montero@diamond.ac.uk

**Keywords:** synchrotron X-rays diffraction, scattering and absorption, field-induced transformations, local *versus* global order, strain and texture analysis

## Abstract

Currently, an intensive search for high-performance lead-free ferroelectric materials is taking place. ABO_3_ perovskites (A = Ba, Bi, Ca, K and Na; B = Fe, Nb, Ti, and Zr) appear as promising candidates. Understanding the structure–function relationship is mandatory, and, in this field, the roles of long- and short-range crystal orders and interactions are decisive. In this review, recent advances in the global and local characterization of ferroelectric materials by synchrotron light diffraction, scattering and absorption are analyzed. Single- and poly-crystal synchrotron diffraction studies allow high-resolution investigations regarding the long-range average position of ions and subtle global symmetry break-downs. Ferroelectric materials, under the action of electric fields, undergo crystal symmetry, crystallite/domain orientation distribution and strain condition transformations. Methodological aspects of monitoring these processes are discussed. Two-dimensional diffraction clarify larger scale ordering: polycrystal texture is measured from the intensities distribution along the Debye rings. Local order is investigated by diffuse scattering (DS) and X-ray absorption fine structure (XAFS) experiments. DS provides information about thermal, chemical and displacive low-dimensional disorders. XAFS investigation of ferroelectrics reveals local B-cation off-centering and oxidation state. This technique has the advantage of being element-selective. Representative reports of the mentioned studies are described.

## 1. Introduction

**Figure 1:**
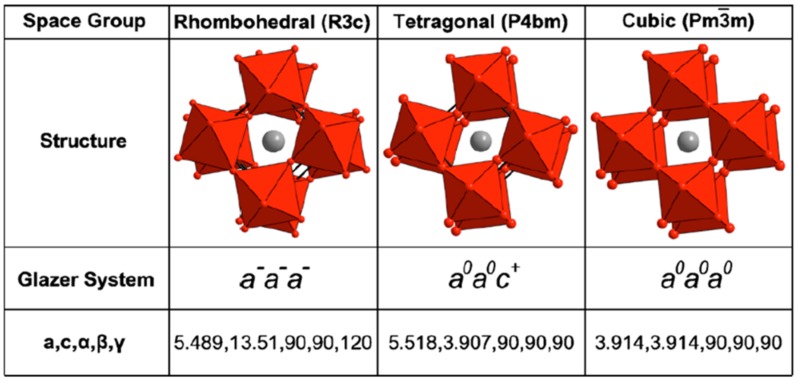
Structural properties of the rhombohedral *R*3*c*, tetragonal *P*4*bm*, and cubic *Pm-*3*m* phases reported for Bi_0.5_Na_0.5_TiO_3_ (BNT). The lattice parameters for the rhombohedral case are given in the hexagonal system. Reproduced with permission from [5].

Materials with ferro- and piezoelectric properties are globally required, respectively, in information technology and in electromechanical transduction. Ferroelectric crystals and ceramics are also piezoelectric. Applied materials science faces today the challenge of finding lead-free ferroelectric materials with high ferro-piezoelectric properties [1]. One of the most common structures of ferroelectric oxides is the well-known perovksite-type arrangement [2], of general formula ABO_3_. It consists of a three-dimensional arrangement of vertex-sharing oxygen octahedra, in between which large A cations are located and whose center is occupied by small B cations. The deformation from the cubic prototype structure, the off-center displacement of the A and B cations and the oxygen octahedra tilting cause the displacement of the centers of positive and negative charges in the unit cell and build-up the spontaneous polarization, *P_s_*, of this type of ferroelectrics. Figure 1 represents the Glazer notation [3] for octahedra tilting in three of the crystal symmetries considered in the investigation of ferro- piezoelectric perovskites. Another important symmetry in ferroelectric crystals is *P*4*mm*, with Galzer notation *a^0^a^0^c^0^* [4]. This latter case looks like *Pm*-3*m* when viewed along the *z* axis.

The history of the understanding of the structural origin of ferroelectricity in BaTiO_3_ (BT) illustrates the importance of the development of new and more powerful crystallographic tools. Up to the 1940s, BaTiO_3_ ceramics were known as intriguing ceramics. Atomic-level explanations for their polarization phenomena were lacking. They were polycrystalline and, in virgin state, isotropic/non-ferroelectric. However, they were capable of being poled and afterward they became ferro and piezoelectric. At the time, the domain structure of BaTiO_3_ was proposed, phase transformations among various crystal systems were reported [6] and the first models for the poling process were suggested [7]. The following important question remained unsolved. Does the BaTiO_3_ tetragonal ferroelectric structure show the necessary inversion symmetry break-down or not? Available photographic XRD methods were not capable of revealing with the required detail the atomic positions in BT [8]. Around that time, the first Geiger-counter measurements of diffracted intensities were essayed at the Philips X-ray laboratories in Irvington (NY). H.T. Evans, from MIT, went to Irvington and examined a BT sample in the recently invented “diffractometer”. His result was the first experimental detection of inversion symmetry break-down in BaTiO_3_ [9]. Further refinement of the BT crystal structure was possible in the following years, thanks to another invention that was just emerging and growing exponentially in impact: the computer [10].

Recent structural descriptions of ferroelectric perovskites often refer to their pseudo-symmetries, due to the existence of local deviations from the global symmetry, arising from the many possible local displacement of the ionic positions and chemical order [11,12].

Regarding the interaction of ferroelectric perovskites with electric fields, it is well known that poling causes increasing orientation of ferroelectric domains as the electric field and time increases until reaching saturation of the remnant polarization, *P_r_*. In this way, poling a polycrystal ceramic creates a macroscopic, non-centrosymmetric ∞*mm* symmetry, which leads to piezoelectric activity. Only the reorientation of the 180° domains, in which the polarization has the same direction and opposite sense at both sides of the domain wall, can undergo a reorientation process without a crystal strain. A non-180° domain reorientation is accompanied by a crystal strain. The remnant polarization of poled ferroelectric polycrystals, *P_r_*, and consequently, their piezoelectric performance, is determined by the existence of domains in which the spontaneous polarization of the crystal is oriented in a limited number of directions according to the symmetry of the crystal structure (tetragonal: *P_r_^max^* = 0.83 *P_s_*, with six <001> equivalent directions of the spontaneous polarization; rhombohedral: *P_r_^max^* = 0.87 *P_s_*, with eight allowed <111> equivalent directions and orthorhombic: *P_r_^max^* = 0.91 *P_s_*, with 12 allowed <110> equivalent directions [13,14]). Those solid solution systems with the so called Morphotropic Phase Boundary (MPB), for which composition there is symmetries coexistence, are desired. This increases the number of equivalent directions of *P_s_* and, consequently, increases the *P_r_* value. It is also possible to increase *P_r_* over the values of the classical randomly oriented ceramics by the processing of textured ceramics oriented in the poling direction. In ferroelectric ceramics, electric field-induced structural transitions have been observed [15,16,17,18,19]. Of particular interest for strain derived applications are those in Bi_0.5_Na_0.5_TiO_3_ (BNT)-based compositions that take place from a weakly polar cubic pseudo- symmetry to ferro-piezoelectrically active phases of lower symmetry.

In comparison with the Evans research on BT, the crystallography arsenal has grown impressively. The resources available to characterize the phenomena of interest include electron microscopy, X-ray and neutron diffraction, Raman spectroscopy and properties measurements, among others. Particularly, presently available synchrotron X-rays beams are millions of times more intense than in those of the 1950s, the types of radiation–matter interaction used today with research purposes is not limited to diffraction, the detection systems show pixel resolution and energy discrimination. Thanks to the mentioned advanced, today, departures from model symmetries (at local and global levels) in real-world materials can be detected with several orders of magnitude higher sensitivity. Finally, the computer capabilities are simply not comparable with the ones that helped decipher the BaTiO_3_ structure. The aim of the present article is to deliver a compact review of the applicability of synchrotron radiation techniques to the study of ferroelectric ceramics. Emphasis will be put on diffraction (well-defined peaks), diffuse scattering and absorption spectroscopy. Lead-free compounds and topics related with crystal symmetry, texture, strain and the effect of electric field on crystal- and microstructures will be highlighted.

## 2. X-ray Diffraction and Scattering

### 2.1. One-Dimensional Measurements

X-ray diffraction (XRD) is a useful, yet complex, tool that has been extensively devoted to studying the evolution of the crystal structure of ferroelectrics under the action of an electric-field using conventional diffraction (CuKα radiation tubes) [20,21,22,23,24] and synchrotron radiation sources [25,26]. It must be noticed that poling and depoling of ferroelectric ceramics can be also achieved by the application of mechanical loads to the sample and there is also extended parallel literature that uses the same structure, strain and texture determination tools and models [27] as those used for the electrical poling, a review of which is outside the scope of this work.

To understand the relevance and uniqueness of the experiments on ferroelectrics using synchrotron radiation, a number of fundamental concepts of powder X-ray diffraction [28] must be bear in mind. First, a polycrystalline sample should contain thousands of crystallites. Such it is the case of a ferroelectric ceramic thin disk or plate with a surface to explore of tens of mm^2^ that is typically constituted by ceramic grains of a few microns size. It should be mentioned that X-ray diffraction provides information from so-called coherent diffraction domains and not from ceramic grains. A coherent diffraction domain is the largest region in three-dimensional space that satisfies the periodic translation of the crystal unit-cell [29]. Grains in ferroelectric ceramics are formed by several crystallites and each crystallite may be formed by different ferroelectric domains. Ferroelectric domains show diverse orientations of the spontaneous polarizations. In a ferroelectric, coherent diffraction domains coincide with ferroelectric domains.

Proper use of XRD data from a polycrystalline sample requires the consideration of all the experimentally observable diffraction peaks.

The most commonly used geometry in laboratory powder diffraction is the parafocussing Bragg-Brentano configuration. In this arrangement, the X-ray tube is fixed and the incident- and diffracted- beam slits are located in points of a circle that is centered on a point at the flat surface of the sample. Divergent X-rays from the source hit the sample at different points on its surface. During the diffraction process, the X-rays are refocused at the diffracted-beam slit. This arrangement provides the best combination of intensity, peak shape, and angular resolution.

Often ferroelectric ceramic thin disks are poled perpendicularly to their faces (“thickness poling” [30]). When analyzing thickness poled disks in this geometry, the electric field effect on the material that is actually probed is limited to the action on a small fraction of crystallites, those contributing to the intersection of the detector scan with the Debye cone, whose *d*_hkl_ is parallel to the electric field. This is a relevant condition since it provides information on the stronger changes that the structure of the ferroelectric material may undergo under the electric field action.

Synchrotron radiation is the brightest light on earth. It is the single most powerful tool available to X-ray crystallographers. X-ray beams are generated by electrons flying at nearly the speed of light in a (roughly) circular loop, guided by strong magnetic fields [31]. Synchrotron radiation is also characterized by its tunability in energy (from E ~ eV to MeV), with a high degree of monochromatization and collimation. In synchrotron radiation diffraction, the wavelengths used are frequently smaller than those of X-ray tubes, which allows scanning to higher Q (=4π·sinθ/λ) with high counting rates. All these characteristics allow the volume representativeness, geometric resolution and statistics of synchrotron experiments to be significantly better than when using X-ray tubes.

Synchrotron intensity is not constant in time. A correction that is often applied consists of a normalization based on the readings of the incident beam monitor.

To take advantage of the high intensity, collimation and monochromaticity of their X-ray beams, synchrotron X-ray diffractometers preferably use the so-called parallel-beam configuration. Synchrotron X-rays do not diverge, unlike those generated from an X-ray tube. This geometry allows spot sizes on the micron scale (producing micro-diffraction) and eliminates the sample displacement systematic error of diffraction experiments. On the other hand, in the Bragg-Brentano arrangement the area bathed by X-rays is larger. In the case of inhomogeneous surfaces, Bragg-Brentano may offer better averaging.

**Figure 2:**
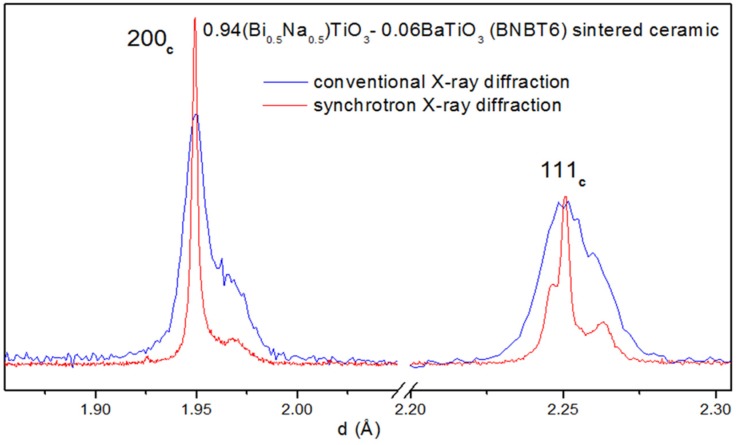
Diffraction peaks from sintered BNBT6 bulk ceramic from a powder flat specimen measured using a conventional Cu-cathode X-ray tube (step 0.05 2θ° and 5 s counting time) and synchrotron radiation at MCX beamline Elettra Sincrotrone, Trieste (Italy) (step 0.005 2θ° and 2 s counting time).

An example of some diffraction peaks measured using a Cu-cathode X-ray tube and synchrotron radiation is shown in Figure 2. The improved resolution makes clear the advantages of the synchrotron X-ray diffraction.

#### 2.1.1. Recent Work on PZT and BaTiO_3_ [12,32]

Structures and phase diagrams of ferroelectric ceramics have been studied since the origin of their discovery. Nevertheless, recent studies with synchrotron radiation and other techniques keep on revealing finer details of classical compositions.

**Figure 3:**
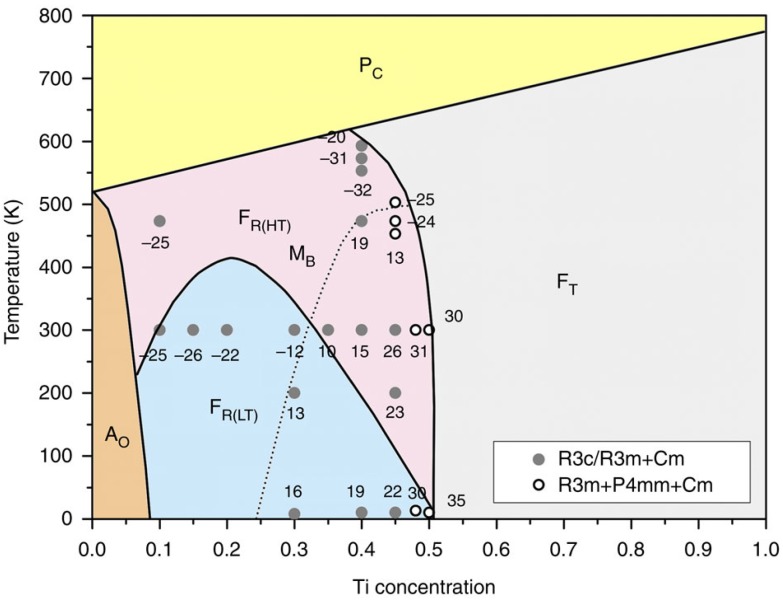
Zhang, *et al*.’s phase diagram for PZT: The crossover between M_B_ and M_A_ structures is marked by a dashed line. The numbers close to the full and hollow small circles represent the deviation angle (in degrees) of the Pb cations shifts, relative to the rhombohedral case. Reprinted with permission from [12].

PbZr_1−*x*_Ti*_x_*O_3_ (PZT) is one of the most important and widely used piezoelectric materials. The study of its local and average structures is of fundamental importance in understanding the origin of its high-performance piezoelectricity. In 1999, using high-resolution synchrotron powder diffraction, Noheda, *et al*. discovered the existence of a monoclinic phase in the Zr-rich region of the PZT phase diagram [33]. In recent years, new significant details of the PZT monoclinic ordering have been observed by the combined use of synchrotron light and neutrons [12,34]. The nature of the monoclinic phase across the Zr-rich and morphotropic phase boundary region of the PZT phase diagram is described in detail in a fresh study by Zhang, *et al.* [12]. Long-range average rhombohedral and both long- and short-range monoclinic regions coexist at all compositions. In addition, a boundary between a monoclinic (M_A_) structure and another monoclinic (M_B_) structure has been established. Both monoclinic structures, at room temperature, belong to the space group *Cm* and in both the Pb cations are located in a {110} symmetry plane. In a rhombohedral arrangement, the displacements of the Pb cations with respect to the cubic structure would be in a <111> direction. In the M_A_ phase, this shift departs from <111> on the way to a <001> direction (symmetry tends to tetragonal). In M_B_, the Pb displacement deviates towards <101> (symmetry tends to orthorhombic). A refined PZT phase diagram (Figure 3) is suggested.

For the monoclinic phase with composition Pb(Zr_0.525_Ti_0.475_)O_3_, at 10 K, the *Cc* space group has been found in a combined synchrotron and neutron powder diffraction study [35].

**Figure 4:**
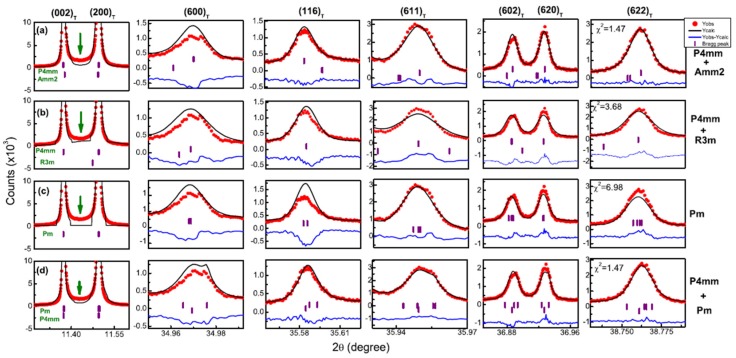
Rietveld fitted synchrotron high-resolution XRD profiles of BaTiO_3_ with models (**a**) *P*4*mm* + *Amm*2, (**b**) *P*4*mm* + *R*3*m*, (**c**) *Pm*, and (**d**) *P*4*mm* + *Pm*. Best fitting model is *P*4*mm* + *Pm*. Reproduced with permission from [32].

Also recently, Kalyani, *et al.* [32] published their finding, based on high resolution synchrotron XRD (Figure 4) and other techniques, that in BaTiO_3_ at room temperature a subtle monoclinic *Pm* phase coexists with the tetragonal *P*4*mm* phase These results propose that BaTiO_3_ at room temperature is within an instability regime, and is structurally more akin to lead-based morphotropic phase boundary systems such as PZT, PMN-PT, PZN-PT instead of PbTiO_3_. The results by Kalyani and collaborators offer a new perspective to understand the anomalous piezoelectric and dielectric responses in poled single crystals and polycrystalline and chemically substituted BaTiO_3_ systems which are increasingly becoming important as lead-free piezoelectric materials.

#### 2.1.2. Texture

Poling of ferroelectric polycrystals is a process that creates a preferential orientation, since it increases the volume fraction of ferroelectric domains with their polarizations as close as possible to the electric field direction. This is done at the expense of the reduction of the volume of the less favorably aligned domains. As a consequence, there is a growth in the intensity of the peaks at the X-ray diffraction pattern corresponding to interplanar distances in parallel with the applied field at the expense of the decrease of others corresponding to interplanar distances perpendicular to the field. The volume of ferroelectric domain alignment can be calculated from the changes in the integrated intensities in the non-poled and poled samples of selected peaks of the diffraction pattern [13,20,21,23,24,25,26,27]. Deconvolution of degenerated peaks for the purpose of peak intensity calculation can be performed by a variety of software [36]. For calculations, correct profile shape functions must be considered [37]. More information related with texture analysis by means of synchrotron XRD will be given in the section about two-dimensional measurements, below.

#### 2.1.3. Diffuse Scattering: PDF Analysis

Diffraction analysis leads to long-range space-and time-averaged high-resolution structure determination via information contained in the diffraction peaks. Material information contained in the background is frequently neglected. However, every point in a diffraction pattern, including the background, contains structural information. Local (static or dynamic) atomic positions deviations from the reference points decrease the peak intensities, in the amount characterized by the Debye-Waller factor. The intensities not appearing in the Bragg peaks contribute to the so-called diffuse scattering. Careful measurement and data processing of (diffraction + diffuse scattering) data leads to the so-called pair distribution function [38] PDF = *G(r)*, containing important information regarding the local environment of atoms in the investigated materials. *G(r)* represents the probability (relative to the average electron density) of finding pairs of atoms separated by the distance *r.* In the particular case of ferroelectrics, differences between local and long-range ordering may be significant.

**Figure 5:**
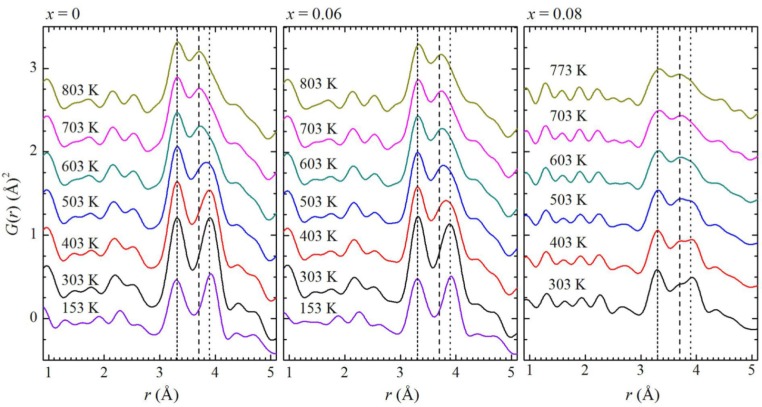
Temperature-dependent X-ray PDF patterns of BNBT for *x* = 0, 0.06, and 0.08. The short-dashed lines (3.3 Å) mark the feature corresponding to A-B distances; the dashed (3.7 Å) and dotted lines (3.9 Å) mark the two components in the feature related to A-A and B-B distances. Reproduced with permission from [39].

**Figure 6:**
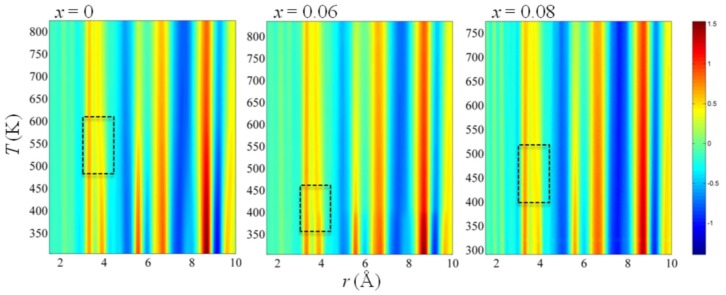
Temperature evolution of X-ray PDF intensities for BNBT for *x* = 0, 0.06, and 0.08; the dashed rectangles mark the temperature ranges of the most pronounced changes in PDFs. Reproduced with permission from [39].

Figure 5 and Figure 6 show the results of a PDF determination by means of an X-ray total elastic scattering experiment [39]. Temperature-dependent powder diffraction data from the (1−*x*)Na_0.5_Bi_0.5_TiO_3−_*x*BaTiO_3_ (BNBT) family (0 ≤ *x* ≤ 0.08) were collected on heating between 120 and 800 K at the BW5 beamline of the DORIS-III facility in Deutsches Elektronen-Synchrotron (DESY) using an incident beam energy of 100 keV (λ = 0.12398 Å), which provided a maximum reciprocal-space *Q* value of 25 Å^−1^. Data processing was performed with program PDFgetX3 [40]. Figure 5 shows the low-*r* range of the BNBTs PDF and Figure 6 shows a wider interval using a color-based representation of PDF intensities.

Some qualitative observations on the cation-cation correlations can be performed from the determined PDFs. The peak at 3.3 Å represents the coupling between the A and B subsystems, basically Bi-Ti distances. These distances remain nearly unchanged both as a function of composition and temperature. The incorporation of Ba affects the A-A and B-B contributions to the PDF around 3.8 Å. The observed variations in mentioned interactions correlate with the enhancement of ferro-piezoelectric properties in the MPB of the BNBT system.

PDF results are consistent with those obtained by Raman spectroscopy.

### 2.2. Two-Dimensional Measurements

The diffraction/scattering research field is changing significantly with the profusion of 2D detectors. Two-dimensional measurements represent a meaningful gain in information from diffraction/scattering experiments. Here, we first describe a few representative 2D detectors and the advantages of using one or another type. Afterwards, we proceed to present a sampler of current research trends using 2D measurements.

#### 2.2.1. Two-Dimensional Image Plate

Two-dimensional Image Plate detectors were initially developed for X-ray Radiography, and then they found applications in X-ray diffraction, in conventional equipment and synchrotron radiation beamlines. Their operating principle is similar to that of a photographic plate emulsion. X-rays impinge on a film containing a phosphor coating which is excited and stores a latent image. Then, the image is “revealed” by scanning the plate with a He-Ne laser beam across it, which produces photoluminescence. The luminescence intensity is read by photodetectors, and the phosphor is erased by exposing it to visible light. The spatial and temporal resolution of the detector depends on the characteristics of the phosphor, laser and associated electronics.

**Figure 7:**
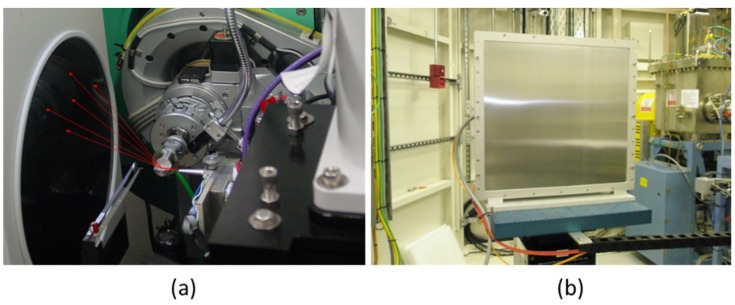
(**a**) MAR345 detector at SSRL beamline 11-3. X-ray trajectory is represented by red arrows; (**b**) Pixium RF4343 detector, covered with Al front plate, at Diamond Light Source beamline I12.

MAR345 image plate detectors are used in synchrotrons for X-ray dispersion and diffraction studies in transmission and grazing incidence geometries. The phosphor consists of a 0.4 mm coating of BaFBr:Eu_2_. An example of its use in grazing incidence XRD to study ferroelectric materials is the work of Torres, *et al* [41], performed at the Stanford Synchrotron Radiation Lightsource (SSRL) Beamline 11-3 (Figure 7a). The use of this kind of detectors for the measurement of crystallographic texture is described in [42].

#### 2.2.2. Charge-Coupled Device (CCD)

The charge-coupled device (CCD) detector consists of an array of metal-oxide-semiconductor capacitors (CMOS). These capacitors detect visible light through a transparent layer of polycrystalline silicon electrodes. Most CCD X-rays detectors record incident photons indirectly through scintillators that detect X-rays and produce visible light. The visible light is then detected by the CCD [43]. The Diamond Light Source (DLS) beamline I12 is equipped with an X-ray imaging camera for radiography and tomography, based on an array of scintillators and CMOS cameras [44].

The Pixium type detector, employed in synchrotron radiation XRD and related techniques, is a CMOS camera with a CsI scintillator. For instance, the Pixium RF 4343 detector conversion of X-ray to visible light is based on a columnar crystalline CsI scintillator array, which offers a high X-ray absorption, due to its high average mass atom number. The visible light is detected by an amorphous silicon photodiode array. In Figure 7b, the detector from the beamline I12 at Diamond is presented. This type of detector is suitable for two-dimensional powder diffraction, Laue-diffraction, total scattering and SAXS experiments [44,45].

#### 2.2.3. Single-Photon—Counting Mode

New detectors have been developed on the concept of single-photon-counting mode [46]. This operation is based on the total absorption of X-ray photon by a scintillator or a semiconductor, which is connected to a cell of collection electrodes. Each cell represents one pixel. The current registered by the pixel is proportional to the energy deposited from the incident X-ray photon. For photons below 100 keV, most events deposit all the energy in a single pixel. In other words, every X-ray photon is directly converted into an electrical signal and counted by the detector system. This is the case of Pilatus detectors [47].

There is a next generation of PILATUS detector, also composed of semiconductor sensor, CMOS readout chip, and readout electronics. X-rays deposit their energy in a pixelated silicon sensor that is typically 320 µm thick [48].

#### 2.2.4. Grazing Incidence 2D-XRD: Texture Analysis

Texture plays an important role in polycrystal physical properties. A detailed discussion about the influence of texture on piezoelectricity can be found in [49].

The main tools for measuring textures are based on neutrons’, X-rays’ and electrons’ diffraction. The neutron sources require access to nuclear (relatively hard to reach) facilities, but provide the most representative results in terms of observed volume [50]. Electron microscopes, in image and diffraction modes, offer grain-by-grain characterizations of texture, but represent local techniques, of low statistical significance [51]. XRD analysis of textures is accessible and leads to average results from significant volumes [52]. With synchrotron radiation the analyzed volumes are greater than with laboratory diffractometers. Particularly, with 2D detection, the exposure times can be shortened dramatically.

Though being a non-ferroelectric, one of the main applications of fibers or nanorods and polycrystalline films of the wurzite type structure ZnO semiconductors lays on their piezoelectric properties.

**Figure 8:**
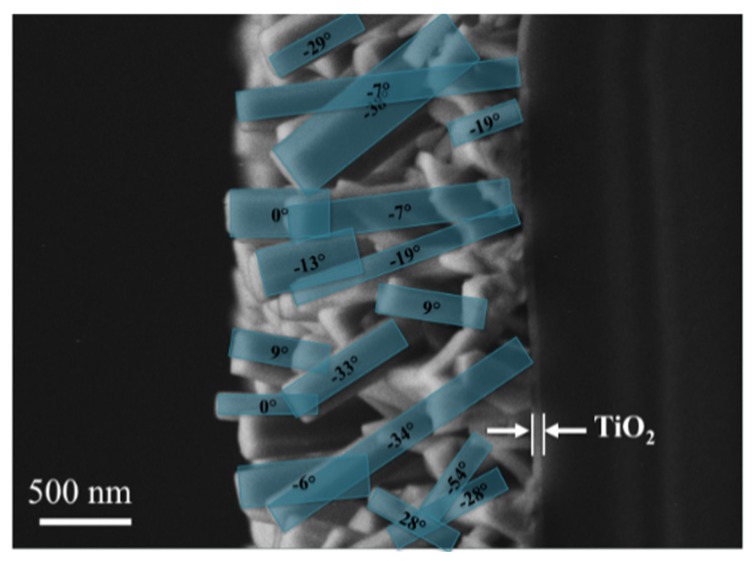
SEM micrograph of textured ZnO thin layer on TiO_2_/borosilicate glass. Cross section view. Image exhibits several ZnO nanorods and their axis inclination from substrate normal. Reproduced with permission from [53].

**Figure 9:**
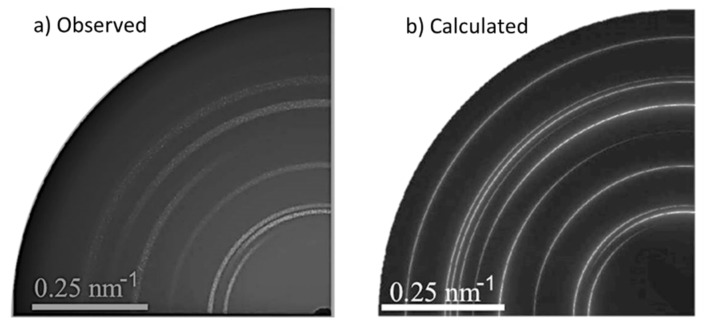
2D-GIXRD patterns of ZnO nanorods: (**a**) as observed at SSRL beamline 11.3 MAR345 detector; and (**b**) showing the texture simulation with program ANAELU [54]. Reproduced with permission from [53].

Figure 8 and Figure 9 exemplify the correlation of electron microscopy and synchrotron 2D-XRD analyses of texture in piezoelectric thin layers [53]. ZnO nanorods were synthesized by aerosol assisted chemical vapor deposition onto TiO_2_ covered borosilicate glass substrates. Figure 8 shows the rod morphology of the ZnO obtained nanocrystals. The SEM evaluation of the nanorods orientation dispersion was performed by analysis of Figure 8.

The sample observed by SEM was also examined at beamline 11-3 of SSRL. The grazing incidence experiment was as shown in Figure 7a. The experimental 2D-XRD pattern is shown in Figure 9a. Diffraction data interpretation was by means of computer-aided modelling. Software package ANAELU [54] was used to simulate 2D diffraction patterns produced by the proposed axially symmetric orientation distributions. In program ANAELU, texture is represented by a Gaussian-shaped inverse pole figure *R*(ϕ) ∝ *exp*–(ϕ/Ω)^2^. ϕ is the angle between reciprocal direction *h* and preferred orientation *h_0_*. Ω characterizes the orientation distribution width. Figure 9b shows the best-fitting modelled output by ANAELU.

SEM and 2D-XRD analyses converged to the conclusion of a preferred growth in [001] direction with a distribution width Ω = (20 ± 2)°.

#### 2.2.5. 2D-Diffuse Scattering: Reciprocal Space Mapping

Real crystals normally show deviations from ideal periodicity. These imperfections reduce the intensity of the diffraction maxima and generate intensity distributions around the reciprocal space nodes. Depending on the nature, dimensionality and importance of these imperfections, the diffuse scattering distributions can form streaks, planes or rods connecting the reciprocal nodes. The diffuse radiation is produced by static disorder (chemical heterogeneities, microdeformations) and/or kinematic disorder (vibration, phonons). The study of these phenomena by means of so-called reciprocal space maps has been significantly favored by the growing capabilities for measuring 2D images of diffuse scattering [31].

**Figure 10:**
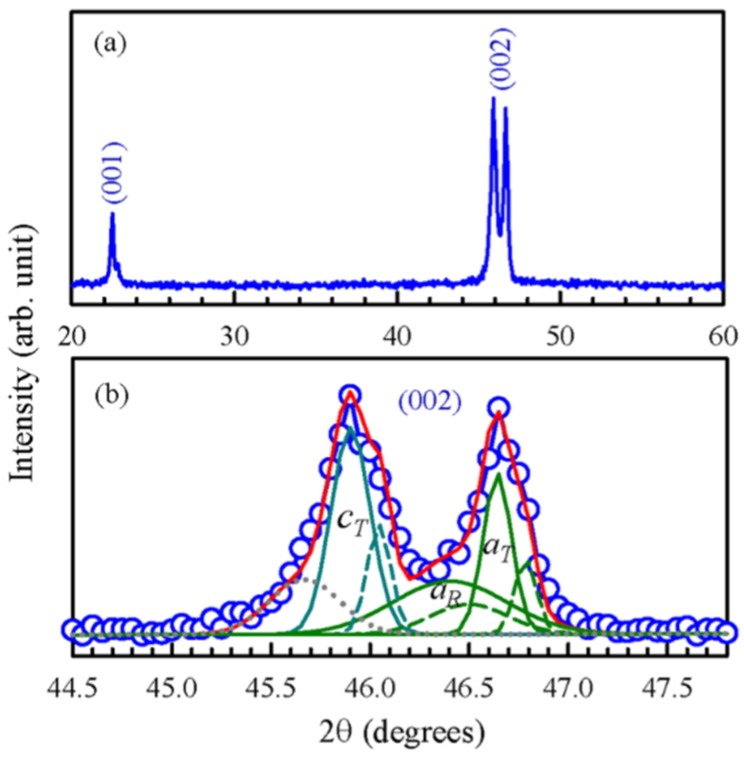
(**a**) XRD spectrum; and (**b**) fits of the (002)c peak for BNB7T crystal. The solid and dashed lines correlate to *K*α_1_ and *K*α_2_ radiations. The red line is the sum of fitting curves. The dotted line is a contribution from the surface layer. Reproduced with permission from [55].

Going back to lead-free ferroelectrics, the work by Chen, *et al.* [55] on 0.93(Bi_1/2_Na_1/2_)TiO_3_-0.07BaTiO_3_ (BNBT7) illustrates the usefulness of 2D mapping of scattered synchrotron X-rays. In the mentioned contribution, textured samples of BNBT7 were obtained from powder at 1300 °C through spontaneous nucleation at the critical temperature by a cooling rate at 1 °C/h. Figure 10a shows the room-temperature XRD obtained in conventional laboratory equipment. The sample shows an intense dual-component [(001) + (100)] texture, as can be seen from the absence of the 110 and 111 reflections. Figure 10b shows in detail the analysis of the reflection cluster at 2θ ≈ 46°. The appearance of the reflection denoted $a_{T}$ demonstrates the presence of a certain population of (100) planes parallel to the sample surface. The $c_{T}$ (002) peak, on the other hand, exhibiting such an important intensity (despite its smaller multiplicity factor) proves that the main texture component represents a [001] polarization out of the plane of the sample. Proper modelling of the observed spectrum requires the consideration of a (scarcely visible) rhombohedral (R) phase and also of a surface artifact to explain a small hump on the low-angle side of the peak cluster (approximately at 2θ ≈ 44°). This hump will be discussed in more detail later.

**Figure 11:**
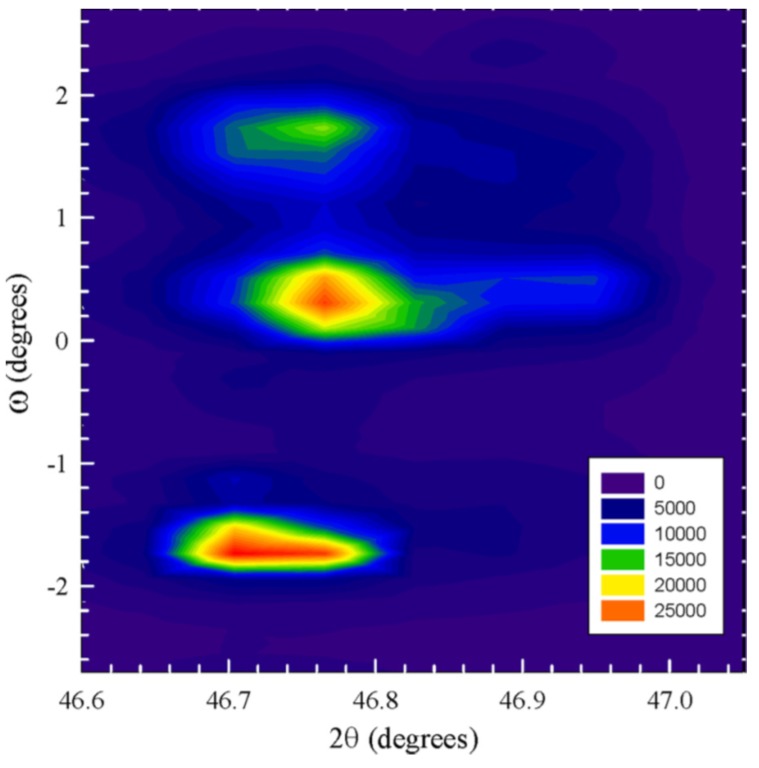
(002)c XRD reciprocal space mapping. The mapping intensity is on a log scale. Reproduced with permission from [55].

Figure 11 shows the synchrotron-measured reciprocal space mapping of the considered peak cluster. The two signals from the T phase and the one from the R component are now clearly displayed by the three contour centers in the two-dimensional reciprocal domain. According to Chen and collaborators, the broad reflections in R and T mapping contours imply nanostructures, which are often expected in relaxor ferroelectric and ferroelastic crystals to accommodate the lattice distortions of different structures. Since nanostructures are usually smaller than the coherent length of X-ray radiation, diffracted waves from individual nanostructures can coherently superimpose and thus broaden the diffraction peaks.

### 2.3. XRD With In-Situ Applied Electric Field

**Figure 12:**
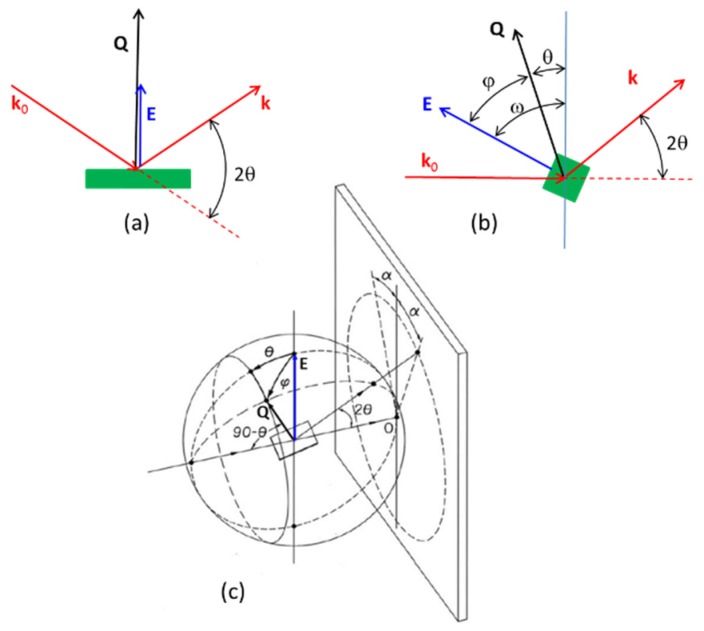
Experimental arrangements for diffraction measurements under electric fields. (**a**) One-dimensional diffraction, symmetric reflection (θ–2θ) with applied electric field perpendicular to sample surface; (**b**) Transmission setup with control of the angle between scattering vector *Q* and field E [69]. The condition ω = 90° represents the case of electric field parallel to the incident beam [68]; (**c**) Generic two-dimensional (2D) detection of diffracted/scattered X-rays. Characteristic directions and angles in the 2D experiment are represented. *Q* is the scattering vector, θ is the Bragg angle, α is the azimuth on the 2D-XRD pattern and φ is the angle between *Q* and E. For α = 0, φ equals the Bragg angle. If θ→0, then φ→ α. Figure 12c adapted from [54].

The development of diffraction experiments under *in-situ* application of electric fields to ceramic specimens at synchrotron radiation facilities has expanded considerably in the last decade [56,57,58,59,60,61,62,63,64,65,66,67,68]. In the literature, one can find a diversity of configurations regarding the relative orientation of the electric field (E) and the diffraction vectors *k*_0_ (incident), *k* (diffracted) and *Q* (scattering) = *k*–*k*_0_. Figure 12 describes some representative experimental arrangements. Table 1 summarizes relations among angles in the presented experimental setups.

**Table 1:** Angles between electric field **E** and scattering vector **Q**.

Setup (Figure 12)	Reference	Angle φ between *Q* and *E*
Symmetric reflection (12a)	Present report	0
	[62]	90°
Asymmetric transmission (12b)	[68] *E* // *k*_0_	φ = ω − θ
	[69] *E* ⊥ *k*_0_	
2D-XRD (12c)	[57,58]	cosφ = cosθ·cosα [54]

The transmission geometry (Figure 12b,c) is used with neutrons and high energy X-rays, possibly with area detectors. In Table 2, we summarize characteristic data of some facilities that are active in the considered field.

**Table 2:** Some synchrotrons active in research on ferroelectricity.

Synchrotron	Beamline	Energy, Wavelength	Diffraction Geometry	Detector	References
HASYLAB, DESY. Hamburg, Germany	B2	25 keV, 0.5 Å	Transmission with parallel electric field	position-sensitive image-plate	[25,56,68]
European Synchrotron Radiation Facility (ESRF). Grenoble, France	ID15B	87.7 keV, 0.14 Å	Transmission with transverse electric field	2D detector (Pixium 4700)	[15,37,57,59].
	ID11	80.5 keV, 0.155 Å	Transmission with transverse electric field or mechanical loads	FReLoN4M area detector	[26,60,64,65]
	BM28-XMaS	9.8 keV, 1,26 Å	Electric field applied parallel to the explored surface	MAR CCD camera	[62]
Elettra Sincrotrone, Trieste, Italy	MCX	13 keV, 0.95 Å	4-circle Huber goniometer	2D detector, MAR345	[70]
Swiss Light Source (SLS). Villigen, Switzerland	MS - X04SA: Materials Science	28 keV 0.443Å	static field in the beam direction	solid-state silicon microstrip, MYTHEN detector	[61]
Advanced Photon Source at Argonne Nat. Lab. Lemont, IL, USA	5-BM-D	65 keV 0.1907 Å	Transmission with transverse electric field	2D detector, MAR345	[63]
Synchrotron Radiation Research Institute (SPring-8). Japan	BL02B1	35 keV 0.35 Å	Transmission, three-axis goniometer for single-crystal orientation	large cylindrical two-dimensional imaging plate (IP) camera	[66,67]

As a remarkable result of the experimental effort carried out with high energy, high brightness and high resolution diffraction measurements, it is now well documented that the response to the electric field, the modification of crystal symmetry, is orientation-dependent. That is to say that the departure from the equilibrium symmetry of each crystallite depends on its initial orientation with respect to the electric field. The origin of this is that ferroelectric domain orientation under the action of the electric field generates anisotropic strains in ferroelectric ceramics [26,64,65] acting locally on the original structure. The orientation-dependent response of the crystal to the field has been also documented by conventional X-ray and neutron diffraction experiments [5,23,71].

In particular, the giant electric-field-induced macroscopic strain characteristic of many BNT-based ceramics [1] has been shown to arise from a combination of the electric-field-induced phase transformation, the induced texture due to ferroelectric domain reorientation, and electric-field-induced crystal strain [58]. The strain associated with the induced phase transformation is volumetric or dilatational, resulting in an equivalent strain in all sample dimensions. Strains induced by both the domain reorientation, or domain wall movement, and field-induced crystal strains, however, are considered to cause shear strains resulting from changes in the spontaneous polarization direction and are therefore mostly volume-conserving, *i.e.*, they mainly lead to local cation displacements [58].

#### Rietveld Analysis of Structures under an Applied Electric Field

The Rietveld method is based on the assumption that the researcher is capable of representing mathematically the structural features of the analyzed system and the effect of such features on the interaction between the material and the X-ray beam. Rietveld programs are constantly evolving with the objective to refine their representation of diffraction/scattering phenomena and thus allow increasingly exact characterizations of crystallographic systems.

For example, homogeneous strains produce peak shifts and inhomogeneous strains cause peak broadening [28]. Full-pattern Rietveld analysis requires consideration of so-called anisotropic peak broadening and peak displacements. This possibility is implemented in currently distributed programs [72]. Examples of Rietveld analysis for refinement of diffraction patterns using a single crystallographic model with anisotropic lattice strains can be found in references [16,73].

The crystallite-orientation dependent action of the electric-field requires careful selection of the starting model for the texture. To characterize real cases, single-component March-Dollase model may result an over-simplification [31]. The superposition of a number of texture components is also considered in available programs. Fitting a texture model to multiple diffraction patterns taken at different sample orientations [74] can avoid some of the limitations of the single-peak fitting and single-orientation texture methods. Also, strain and texture can be co-refined simultaneously [75]. This means that electric field induced piezoelectric strain and the full orientation-dependent crystal structure of ferroelectric ceramics could also be refined under *in situ* electric fields if sufficient three-dimensional diffraction data could be measured using 2D detectors. Indeed, some facilities are available and there is nowadays a general trend of development of synchrotron sample environments to study the next-generation of field-driven device physics [69,76,77].

A useful approach for the structure refinement from single X-ray diffraction patterns obtained for ceramic bodies [78] is adding to a starting model—representing the prevailing symmetry—a number of secondary phases to simultaneously account with coexisting equilibrium symmetries. Such is the case of some BNT-based ceramics even in the unpoled state [11,79,80]. For BNT and BNT-based and other lead-free ferroelectric ceramics, this strategy has been widely used [70,81,82,83,84,85,86,87].

A single diffraction pattern from a θ–2θ scan using a flat specimen can be related with 2D experiments. For the case of high-energy synchrotron radiation (small wavelength, small Bragg angle), the symmetric 1D experiment provides approximately the same information as the data recorded in a transmission 2D experiment for α = 0 (Figure 12c). The 1D experiment is more sensitive to surface effects at low Bragg angles, which explains why one might do one or the other depending on if surface effects are the focus of the experiment. In literature, one can find informative studies that carry on Rietveld structural refinement of single conventional or synchrotron X-ray diffraction patterns with flat specimens and coupled θ–2θ scans, of ferroelectrics after the application of electric field [70,73,78,81,82,83,84,85,86,87,88,89].

The validity of such a structure refinement resides in that this represents the simplest but a sufficient interpretation of the obtained diffraction data. When applied consistently to a given diffraction geometry [78,89], it does offer a method for comparing structures in a series of samples from single diffraction patterns, which allows understanding electric field effects and their relationship with the functional properties of the ferroelectric ceramics.

## 3. X-ray Absorption Fine Structure Spectroscopy

### 3.1. General

X-ray absorption fine structure (XAFS) spectroscopy is another useful synchrotron technique for understanding ferroelectric systems and many other materials. The considered phenomenon is the amplitude modulation of the X-ray absorption coefficient in energies near and above an absorption edge of a given chemical element. It appears only when atoms are in crystals or molecules. Experimentally, it involves the measurement of the X-ray absorption coefficient as a function of photon energy above the K or L absorption edges of a study atom. These experiments require tuned synchrotron radiation. The absorption edge energies used for XAFS are in the range of tenths to tens keV.

**Figure 13:**
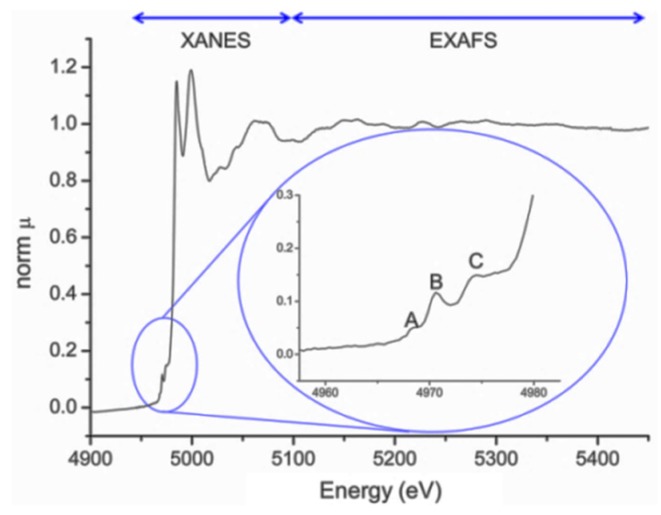
Normalized XAFS spectrum of the Ti K edge of the centrosymmetric perovskite LaFeNiTiO_3_, recorded at Stanford Synchrotron Radiation Lightsource. XANES and EXAFS regions are presented. The inset corresponds to the pre-edge of XANES zone. The meaning of peaks A, B, and C is given in the text.

The observed XAFS effects are conventionally divided into two energy zones, nearby the absorption edge (so called X-ray Absorption Near Edge Structure—XANES) and the extended one (Extended X-ray Absorption Fine Structure—EXAFS), about 50–80 eV above the absorption edge. An example of an experimental XAFS spectrum of an oxide material is presented in Figure 13. These regions contain different information on the chemical states of the absorbing element and neighboring atoms. Both phenomena offer information on the local atomic coordination to distances normally of 0.5 nm and sometimes until 0.8 nm. XANES offers information on oxidation state of the absorbing element. EXAFS allows the determination of interatomic distances, coordination numbers and degree of thermal and/or structural disorder of the local structure (first few atomic coordination shells) surrounding the absorbing atom. The information is averaged over all absorbing atoms of the same type in the studied sample. The XAFS phenomena are consequence of the X-ray photoelectric effect in the studied material, and the further behavior of the scattered photoelectron. These techniques can be applied to almost any element of the periodic table regardless crystallinity state (samples can be amorphous or crystalline) or concentration, *i.e.*, XANES and EXAFS limits of detection are 10 ppm and 100 ppm, respectively.

The XAFS spectrum *χ*(*E*) is defined phenomenologically [90] as the normalized, oscillatory part of the x-ray absorption coefficient above a given absorption edge, *i.e.*,

(1) χ(*E*) = [µ(*E*)–µ_0_(*E*)]/Δµ_0_,

where µ_0_(*E*) is the smoothly varying background absorption function, that would occur if the absorbing atoms were far apart (so-called “bare atoms”) and Δµ_0_ is the height of the absorption edge as normalization factor. Further, the XAFS spectrum is transformed for obtaining the information it provides.

Descriptions, fundamentals, experimental methods, data reduction and interpretations of XAFS is well documented in books [91,92,93] and in many articles. The application of XAFS in ferroelectricity studies is also well supported. The reader is referred to the text recently published “X-ray Absorption Fine Structure Applied to Ferroelectrics” [94], where the foundation and merits of the method are described. The content includes the significant XANES effects for the study of ferroelectric materials, the experimental methods and XAFS spectra processing. It offers a review representing XAFS applied to ferroelectrics, from the explanation of the displacive or order-disorder nature of the materials PbTiO_3_ and BaTiO_3_, to those dedicated to relaxors and Aurivillius oxides.

XAFS spectroscopy helps in studying ferroelectric materials by analyzing experimental results in both XANES and EXAFS regions. In the EXAFS region the information consists of interatomic distances, coordination numbers and a parameter related to the disorder of the local structure around the absorbing atom*.* The EXAFS averaged spectrum of each absorption edge of a study element is processed to retrieve the dependence of absorption coefficient on the photoelectron wave number and then to generate a function related to the radial distribution function around atoms of the given element. Particularly interesting is the interpretation of experiments in which absorption edges of two or more different elements are measured. In these cases, the whole set of modeled coordination numbers, interatomic distances and Debye-Waller factors must fulfill compatibility conditions that lead to auto-consistent global solutions [95].

Here, we will briefly outline the observed features on the XANES spectra and the bases of the involved phenomena.

**Figure 14:**
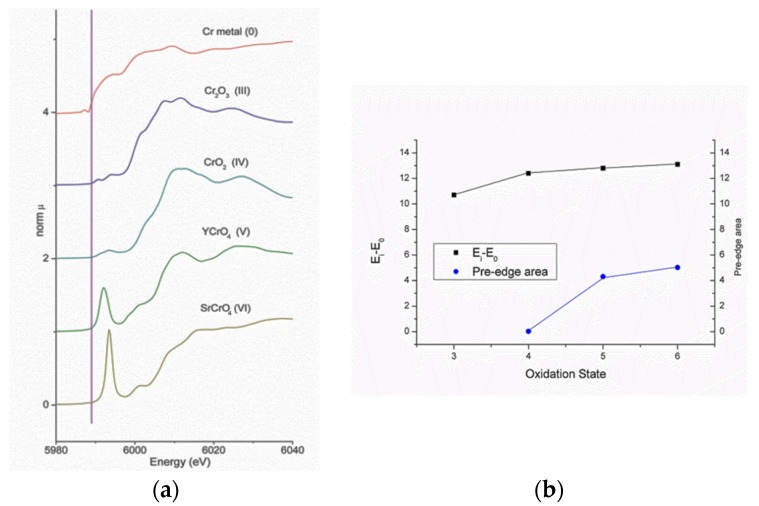
(**a**) XANES spectra of Cr compounds of increasing Cr oxidation states. The vertical line is the position of the first inflection (*E*_0_) in the edge on Cr metal spectrum. Spectra are vertically shifted for clarity; (**b**) Oxidation state dependence of the main absorption edge position (first inflection—*E*_i_) and pre-edge peak area from the spectra presented at (a) [100].

Many ferroelectric materials include in their structure transition metal elements. The effect of the transition metal oxidation state and the cation site symmetry in the XANES spectra is illustrated in Figure 14. When the oxidation state rises, there is a shifting of the absorption edge towards increasing energies. This increase in oxidation state is often associated with cation sites that deviate from the centrosymmetric, and transitions at energies below the absorption edge (“pre-edge transitions”) intensify. These phenomena have been reported for almost all transition metals [96,97,98].

The pre-edge feature exists as a result of the photoelectron transitions from 1*s* state to unoccupied excited states. In the inset of Figure 13 are shown the characteristic peaks of the pre-edge zone, depicted as A, B and C. The nature of the pre-edge transitions has been carefully studied by Vedrinskii, *et al.* [99]. The peak A corresponds to a quadrupole transition of low intensity. Peak B is related to a transition of the photoelectron from the 1*s* state to 3*d* states. These transitions in the metal state (oxidation 0) do not take place because the metal atoms are in a very symmetrical environment and the change in the *l* quantum number is 2 (quadrupole transition). However, for tetrahedral or distorted octahedral geometries of increasing oxidation states, *3d* state mixes with the 2*p* ligand oxygen and the transition *s-p* (dipole transition) is not hindered, resulting in the increasingly intense peak observed in the XANES spectrum. The intensity of the peak C is attributed to transitions of the photoelectron from the 1*s* state to 3*d* levels of neighboring octahedra.

Vedrinskii, *et al.* [99] have obtained the Expression (2) to evaluate the displacement of the titanium cation from the centrosymmetric position. The area under the peak B 〈*I_B_*〉 is directly proportional to the mean square displacement 〈δ^2^〉 of Ti and inversely proportional to the power of the lattice parameter *a* of the perovskite cell:

(2) $$\langle I_{B}\rangle =K\;\frac{\langle \delta^{2}\rangle }{a^{5.5}}$$

This expression and the overall explanation given by [99] of pre-edge transitions play a significant role in the application of XAFS to the study of the ferroelectric perovskite-like structure.

The nature of the pre-edge transitions has been carefully studied also by [101,102,103]. In particular, Yamamoto [103] observed that the pre-edge peaks in K-edge spectra for transition metals with 4*d* electrons, and LI-edge of 5*d* elements should be analogous to those for 3*d* metals.

Technological interest in ferro-piezoelectric ceramics requests characterizing their structures and interactions at the atomic coordination scale. The studies referred to below, aimed at understanding the local-structure basis of polarization, show sustained interest in ferroelectric prototypes (PbTiO_3_, BaTiO_3_, PZT) as well as in novel highly demanded materials (lead-free ferroelectrics).

### 3.2. PbTiO_3_-Based Ceramics Studied by XAFS

A case is seen in the work of Mesquita, Michalowicz and Mastelaro [104], which presents the role of the replacement of Pb by La in the local and electronic structure of PZT and its influence on the ferroelectric behavior. For this purpose, XANES spectra of Ti and O of ceramics Pb_1−*x*_La*_x_*Zr_0.4_Ti_0.6_O_3_ (PLZT100*x*) with compositions *x* = 0, 0.05, 0.11, 0.12, 0.13, 0.14, 0.15, 0.21 were studied. Figure 1 in their work shows the spectra resulting from the Ti K-pre-edge. It shows the feature peaks A, B and C, similar to those in Figure 13. The feature B corresponds to the dipole transition occurring in transition metals surrounded by oxygens in distorted symmetry. By determining the peak areas designated as B, and applying a simplified version of Equation (2), reported in [101,102], the authors [104] assessed the Ti cation displacement from the centrosymmetric structure, descending from 0.42 Å for *x* = 0–0.28 Å for *x* = 0.21. In that paper, they conclude that the replacement of Pb by La leads to the ferroelectric phase transition from normal to relaxor. Other works devoted to study PbTiO_3_ and PZT by XAFS are [105,106].

### 3.3. BaTiO_3_-Based Ceramics Studied by XAFS

**Figure 15:**
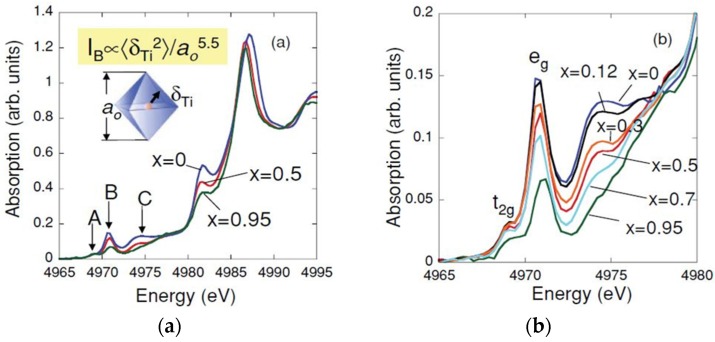
Ti K-absorption edge XANES spectra from Ba(Ti,Zr)O_3_ samples studied in [107]. (**a**) Spectra for selected compositions, indicating the pre-edge features A, B and C. Expression (2) from [99] and structural parameters are also shown; (**b**) Ti pre-edge features for all measured compositions, where systematic variation in areas of features B and C is especially noteworthy. Reprinted from [107]. Copyright by the American Physical Society, 2011.

**Figure 16:**
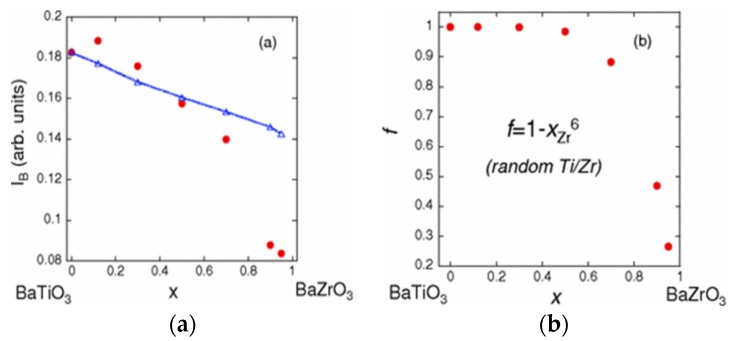
Compositional dependence of the area of pre-edge peak B in Ti K-edge XANES spectra (red circles). (**a**) The blue dashed line/triangles indicate the peak B area reduction according to the *a^5.5^* term in Equation (2) if *a* values are equal to the XRD lattice parameter; (**b**) Calculated compositional dependence of the fraction *f* of Ti cations having one or more Ti as a next-nearest neighbor for a random distribution of Ti and Zr cations occupying the sites B in perovskites. Reprinted figure with permission from [107]. Copyright by the American Physical Society, 2011.

The paper of Levin, *et al.* [107] remarkably presents the use of feature B in the pre-edge region of Ti K-edge to study the local structure of the solid solutions type Ba(Ti,Zr)O_3_ in different concentrations. Ti and Zr cations behavior is characterized by the measurement of Ti K-edge XANES and Zr K-edge EXAFS. It should be noted that is not possible to acquire the Ti K-edge EXAFS spectrum when Ba is present in the sample, because the Ba LIII-edge energy is greater but close to that of Ti K-edge and the Ba absorption edge signal is much larger than that of the Ti in that region. Figure 15 shows the XANES spectra presented in [107]. The article uses the Expression (2) [99] to evaluate the displacement of the titanium cation from centrosymmetric position, as shown in Figure 15a. They compute the structures by density-functional theory (DFT) and use XRD data for clarifying how the Ti cations are locally ordered in dilute systems, and compare these results to those obtained from the peak B area of the Ti pre-edge XANES. The comparison is presented in Figure 16. The DFT and XRD results presented in [107] suggests that when Ti or Zr cations occupy the B site in a relatively isolated environment, the perovskite host lattice somewhat expands but the area I_B_ of the pre-edge peak B (red circles in Figure 16a) decreases, that is, the B cation remains nonpolar. On the contrary, when there are neighboring Ti ions, these atoms are displaced off center, especially in the 〈111〉 direction. The pseudocubic cell with displacements closer to the 〈111〉 directions does exhibit stronger Ti 3*d*–O 2*p* hybridization and, consequently, larger areas of the pre-edge feature B. Authors [107] concluded that relatively large concentrations of the same type B atoms are needed to create enough Ti–Ti or Zr–Zr pairs for ensuring a perceptible B type polarization.

### 3.4. The (Bi_0.5_Na_0.5_)TiO_3_ Family Studied by XAFS

**Figure 17:**
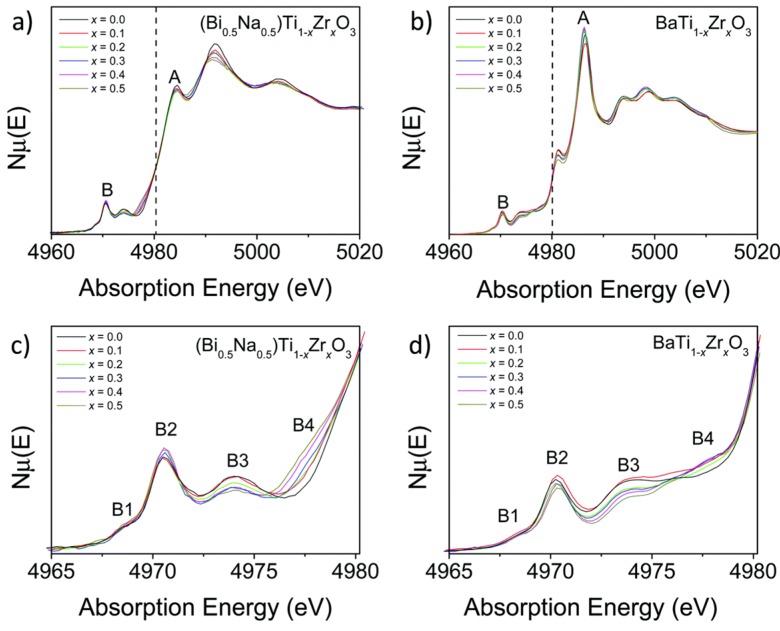
The Ti K-edge XANES spectra of representative members of the (**a**) (Bi_0.5_Na_0.5_)Ti_1−*x*_Zr*_x_*O_3_; and (**b**) BaTi_1−*x*_Zr*_x_*O_3_ solid solutions. Major features of the Ti K-edge pre-edge region of the (Bi_0.5_Na_0.5_)Ti_1−*x*_Zr*_x_*O_3_ and BaTi_1−*x*_Zr*_x_*O_3_ solid solutions are highlighted in (**c**) and (**d**). Reproduced under a Creative Commons Attribution 3.0 Unreported License.

XAFS spectroscopy has also been applied to the study of BNBT’s family, with the aim of studying the effects of substitution of Ti by Zr [108], and the replacement of sodium by potassium [109]. The paper by Blanchard, *et al.* [108] devotes marked interest, again, in the behavior of the Ti K-edge and Zr LIII-edge XANES spectra, focusing on five important photoelectron transitions. Figure 17, extracted from the article, presents the Ti K-edge XANES spectra of the (Bi_0.5_Na_0.5_)Ti_1–*x*_Zr_*x*_O_3_ for *x* = 0.0, 0.1, 0.2, 0.3, 0.4 and 0.5. For comparison, Figure 17 shows also XANES spectra for BaTi_1–*x*_Zr_*x*_O_3_ in the same composition interval, similar to those studied in [107].

The first results noticed from XANES spectra in Figure 17 is that the doping of both ceramics types with Zr does not induce a shift in the energy of the main jump of the Ti K-edge. Nonetheless, Zr doping does have influence in the increase or decrease of dipole-nature peak in the pre-edge, denoted by B2 in Figure 17.

The work [108] associates the decrease in B2 intensity in BaTi_1−*x*_Zr*_x_*O_3_ with the trend of Ti atoms to locate in a more centrosymmetric position inside the octahedron, while the (Bi_0.5_Na_0.5_)Ti_1−*x*_Zr*_x_*O_3_ ceramic displays the opposite trend. The changes in the intensities of the peaks B3 and B4 when Zr concentration increases are attributed to transitions of the photoelectron from the 1*s* state to 3*d* levels of neighboring octahedra, and then their behavior suggests that Ti^4+^ and Zr^4+^ cations are randomly distributed and there is no clustering of TiO_6_ and ZrO_6_ octahedra.

The XAFS analysis of the BNBT group of compounds, derived from BNT, is analyzed in detail below.

## 4. Case Study: The BNBT System

### 4.1. Introduction

We devote our last section to the intensively studied system (1−x)(Na_0.5_Bi_0.5_)TiO_3_–*x*BaTiO_3_, commonly denoted (1−*x*)BNT–*x*BT or BNBT100*x*. Despite the extended literature on the structure–property correlation in this family of materials, there exists nowadays some discussion about the equilibrium structures at room temperature of the compositions near and at the MPB, especially in the 0.04 < *x* < 0.07 interval, as well as about their changes with the electric filed. We summarize representative publications and divulge our results and considerations.

**Figure 18:**
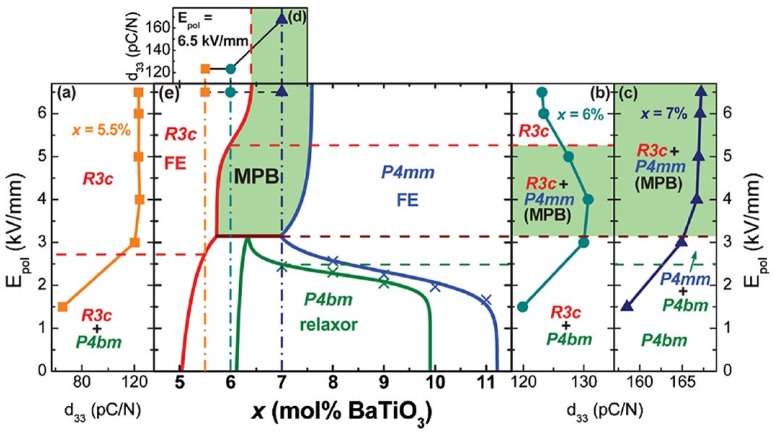
Electric field modulated BNBT phase diagram. Ferroelectric *R*3*c*, relaxor *P*4*bm* and ferroelectric *P*4*mm* regions in the composition-electric field space are identified. Plots of the piezoelectric coefficient *d*_33_ as function of the electric field intensity, for selected compositions, are included. Reproduced with permission from [110].

As a starting reference, we reproduce in Figure 18 the BNBT room temperature phase diagram, with consideration of electric field application, given by Ma, *et al* [110] and obtained by Transmission Electron Microscopy (TEM) and Selected Area Electron Diffraction (SAED) techniques.

In the diagram, the following elements of the MPB are worth being noticed. For *x* = 0.04, the rhombohedral *R*3*c* symmetry (ferroelectric) remains independent of the applied electric field. For *x* = 0.06, in absence of electric field, a pseudocubic global symmetry, weakly polar at the nanoscale and consisting of polar nanoregions of tetragonal *P*4*bm* symmetry (ferrielectric) embedded in a cubic matrix and a global rhombohedral *R*3*c* phases coexist, with predominance of the pseudocubic component. Under an intermediate electric field, a different tetragonal phase, ferroelectric *P*4*mm*, showing domains with lamellar morphology substitutes the *P*4*bm* fraction. If an intense electric field is applied, fully poled sample shows only rhombohedral *R*3*c* symmetry. For *x* = 0.07 and zero electric field, the sample shows only the same pseudocubic global symmetry as for *x* = 0.06. If an electric field is applied, *P*4*mm* appears in the range of moderate applied field and *R*3*c* appears for intense applied field, where a mixture of *P*4*mm* and *R*3*c* exists.

Diverse variants to the Ma, *et al* representation have been published [15,19,68,70,83,111]. In the following, we revisit the 0.04 < *x* < 0.07 interval. Following the general purpose of the article, diffraction and absorption techniques are considered.

### 4.2. Diffraction

We comment first the *x* = 0.04 and *x* ≥ 0.07 cases, where our proposed models are similar to those just revised, and then the *x* = 0.06 composition, where the diversity of models in the literature is larger.

For BNBT4, the possibility of a monoclinic *Cc* structure has been proposed [83]. At the time of this writing, this model had not been confirmed by other publications. The work [70] agrees with Ma, *et al.* and delivers a quantitative characterization of the considered *R3c* structure.

A summary of last-mentioned data is included in Table 4 and Table 5, below.

The particular case of a poled BNBT7 ceramic has been studied in detail by [15]. A synchrotron high energy XRD study showed the splitting of the cubic perovskite 200 peak and its evolution with the α angle (Figure 12). In this study, the considered structures were referred to as pseudo-cubic, for zero E-field, and tetragonal *P*4*mm* for intense electric field. Anisotropic lattice strains were detected.

Last mentioned experiment is compatible with the one by Ma, *et al.* It is worth explaining here the relationship between the *Pm-*3*m* cubic, the *P*4*bm* and the *P*4*mm* tetragonal models.

The cell geometry of the considered *P*4*bm* model of the polar (ferrielectric) nanoregions of unpoled BNBT6 and BNBT7 is also the high temperature symmetry for BNT. This is significantly different from the *P*4*mm* model (ferroelectric) associated with poled BNBT7 (and BNBTs with *x* > 0.10). In *P*4*mm*, TiO_4_ tetrahedra are stretched and cell parameter *c* is measurably larger than *a.* Consequently, 00*l* diffraction peaks split from *h*00 ones. In *P*4*bm*, tetrahedra are tilted (*a^0^a^0^c^+^*, in Glazer notation) and cell parameters are such that the positions of diffraction peaks coincide with the ones generated by the cubic *Pm-*3*m* perovskite structure. No peak splitting occurs. The differentiating feature of the unpoled BNBT6 and BNBT7 *P*4*bm* diffraction pattern is the appearance of superlattice peaks. These peaks are extremely weak. They are practically non-observable by X-ray (including synchrotron light) diffraction. The electron microscopy and neutron diffraction experiments by [110,111] revealed the mentioned superlattice peaks.

**Figure 19:**
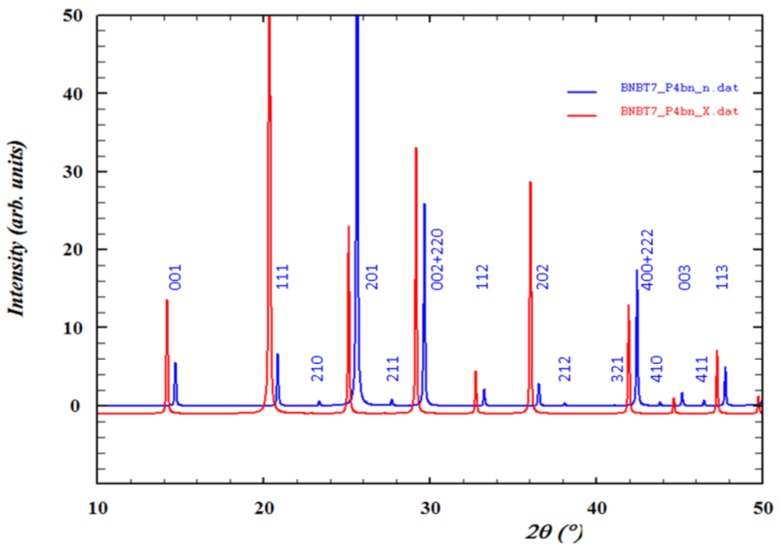
X-ray (red) and neutron (blue) diffraction patterns for tetragonal *P*4*bm* BNBT. Wavelength: 1 Å. Tetragonal indexing of peaks. Indexes in the lower level correspond to superlattice reflections.

Figure 19 shows the X-ray and neutron calculated diffraction patterns of BNBT7, modeled with the Kitanaka [111] *P*4*bm* model. The patterns have been shifted to facilitate observation. Notice the mentioned details.

**Figure 20:**
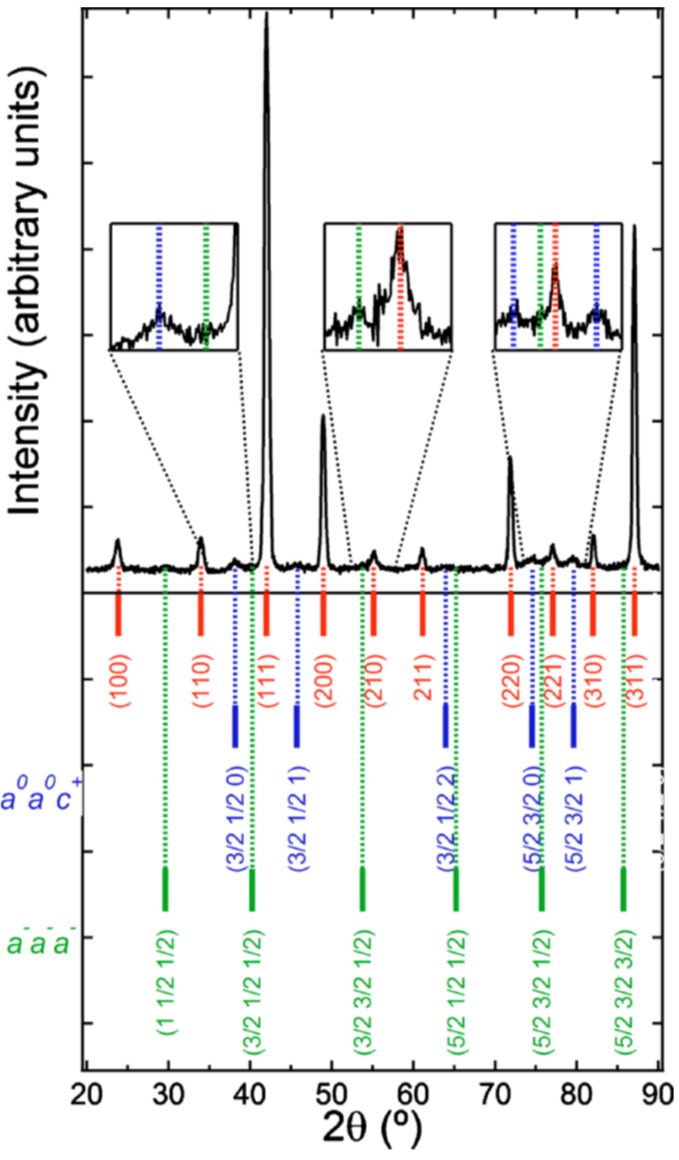
High resolution neutron powder diffraction pattern collected at a wavelength of 1.622 Å. Peak indexing shows contributions from the *a*^0^*a*^0^*c*^+^ (*P*4b*m*) and *a*^−^*a*^−^*a*^−^ (*R*3*c*) tilt-systems, respectively. Reproduced with permission from [5].

By means of XRD experiments, it is difficult to distinguish tetragonal *P*4*bm* from cubic *Pm-*3*m* symmetry [112]. On the one hand, the conjunction of the neutron diffraction [5] (Figure 20), TEM and SAED information [110,113,114,115] unambiguously document the existence of polar phases at the nanoscale. On the other hand, the dielectric relaxor characteristics [116], of BNBT6 and 7 ceramics must be taken into account to select the coexistence of nanoregions of tetragonal *P*4*bm* symmetry (ferrielectric) embedded in a cubic *Pm-*3*m* (non-polar) matrix as the equilibrium room temperature symmetry for these two ceramics.

Some early reports using conventional XRD, see, for example, [19], on poled BNBT6 and BNBT7 ceramics associated the relatively complex profile of *h00* peaks (cubic indexing) as the characteristic 00*l*/*h*00 *P*4*mm* tetragonal peak splitting for BNBTs with *x* > 0.11 (Figure 18).

Recent neutron, electron and XRD synchrotron experiments [68] characterized the structural differences between BNBT6, and BNBT7. By extension of the currently accepted peak splitting association with *P*4*mm* tetragonal symmetry, this work reports that BNBT6, in the unpoled condition, shows a coexistence of rhombohedral *R3c* and tetragonal *P*4*bm* global ferroelectric symmetries. Upon application of an electric field, the *P*4*bm* component evolves to *P*4*mm*.

**Figure 21:**
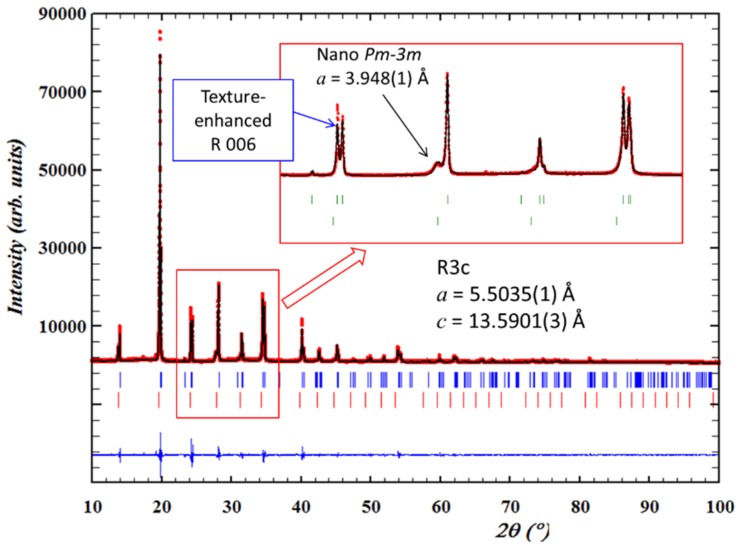
Synchrotron X-ray diffraction pattern and Rietveld analysis of a hot-pressed and recrystallized BNBT6 ceramic flat specimen after poling with electric field perpendicular to the explored surface. Reliability factors: *R*p = 5.63, *R*wp = 8.06. Inset shows some relevant peaks of the ceramic perovskite structure consisting on a rhombohedral *R*3*c* symmetry and a nanosized cubic symmetry, associated with the humps that appear at the low angle side of the main perovskite peaks.

Alternatively, a Rietveld analysis of synchrotron X-ray diffraction patterns [87] of unpoled BNBT6 ceramics from nanopowders [117] led to the conclusion that the relatively complex structure of *h00* peaks of this material involved the coexistence of three components. According to these authors, the analyzed BNBT6 ceramic shows a major cubic *Pm-*3*m* phase, polar at local level as revealed by XANES, that produces the most intense, sharp perovskite peaks. This model is in agreement with both early XRD studies [112] and recent TEM and SAED studies [110,113,114]. Besides this primary phase, two other components were identified. The presence of a rhombohedral *R*3*c* phase was demonstrated by the appearance of the characteristic 113 (hexagonal notation) weak peak. The third phase was ascribed to significantly wide peaks (See Figure 21), humps, appearing at the low-angle side of the sharp and intense cubic *Pm-*3*m* global structure peaks. It was modeled as nano-sized crystallites (~20nm).

Due to the dielectric relaxor behavior of this BNBT6 ceramic [116], related with the existence of polar nanoregions (PNRs), humps [87] have been associated with PNRs, presumably of local *P*4*bm* symmetry [87]. PNRs were observed by TEM in BNT6 [110,114] and have been also detected in PLZT, PMN-PT, PZN and PMN by force microscopy [118] and neutron radial function distributions (RFDs) [119].

Recent literature [120] has pointed that, as the assumed size of the PNR is comparable or below the coherence length for X-rays and neutrons, the information about PNRs obtained by diffraction/scattering is of limited accuracy.

To elucidate the applicability of the three phases’ interpretation of the BNBT6 (and close compositions at the MPB) synchrotron XRD patterns [87] as an alternative to the *P*4*mm* tetragonal symmetry, the nature of the low-angle side humps in BNT-based ceramics [70,78,82,83,84,85,87,121] and single-crystals [122] and their dependence with respect to the main perovskite intense peaks must be clarified.

We report here the Rietveld analysis of synchrotron X-ray diffraction patterns of poled BNBT6 ceramics, identical to those unpoled samples of our previous study [87]. Poling has been performed by a saturation electric field, applied *ex-situ*, in the thickness direction.

Poled bulk ceramics after electrodes soft removal and crushed powder from these samples were studied. Materials handling is described in [123].

Since the electric-field action on the structure and properties of constituent crystallites has been found irreversible at room temperature for this composition, both specimens are expected to have the same crystal structure. The experiment aimed to shine light on the nature of the component ascribed to the repeatedly observed humps in BNT-based ceramics [70,78,82,83,84,85,87,121] and single-crystals [122].

High Q and high counting statistics synchrotron diffraction experiments were carried out using a coupled θ–2θ scan (from 10° to 100° 2θ) with flat specimen at the four circle diffractometer of the MCX line at Elettra Sincrotrone [124] using an X-ray beam of 13.048 keV (λ = 0.9500 Å). Longer data collection times in the 2θ final segment allowed measuring the weak signals of this region with statistical errors σ*_rel_* ≈ 2%. Prior to any calculation, the different segments are normalized considering the measuring conditions and readings of the incident beam intensity monitor.

Figure 21 shows the pattern obtained for the poled ceramic and the corresponding Rietveld analysis (program Fullprof [125]) plot with reliability factors. The pattern was refined for the coexistence of a global rhombohedral *R3c* phase and nano-sized crystallites, modeled as cubic (*Pm-*3*m*) symmetry.

Comparison of Figure 21 with the corresponding XRD patterns in [87] shows that, under polarization, the global rhombohedral *R*3*c* phase increases at the expenses of the weakly distorted pseudocubic/tetragonal (*Pm-*3*m/P*4*bm*) symmetry, a major component in the non-poled sample. This scheme follows the non-ergodic relaxor to ferroelectric field-induced phase transition in BNBT6 [116,126,127].

It must be noticed that, in our studies, humps take place both in hot-pressed and recrystallized as well as in sintered ceramics [128] and both in unpoled [87] and poled samples (Figure 21). Consequently, we can discard the origin of this feature of the pattern as due to texture or other processing parameters of a given ceramic specimen.

When comparing the results of the poled ceramic with our previous results in non-poled ceramic samples, it is noticeable that the integrated intensities of the humps remain virtually unchanged after poling. If the humps were associated with the diffraction peaks of the major perovskite component, for example, as the tetragonal 002/200 doublet, such humps should increase with polarization, as a sign of non-180° domain reorientation. This effect has not been observed.

**Figure 22:**
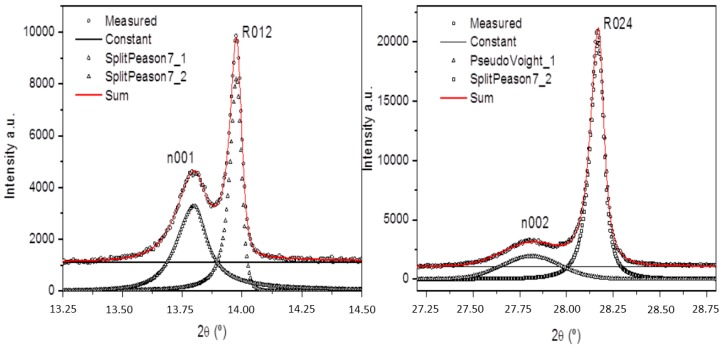
Magnification of two peaks showing the 2θ angular dependence of the intensity of the humps. Peak fitting was carried out using the software described in [36].

It must also be noted that the relative areas of the humps with respect to those of the rhombohedral peaks (Figure 22) decrease as the 2θ angle increases in the poled sample. This suggests that humps are the signature of a surface-related component. We are exploring a volume of a depth of few tenths of ceramic grains. Humps are the manifestation of a structural feature at the surface of that explored volume. Experiments in modified lead titanate and lead zirconate-titanate ceramics showed that there exists a near-electrodes surface layer (few microns) unaffected by the electric poling field due to space charge effects and domain-wall blocking mechanisms [129]. Therefore, humps in non-poled and poled samples could well have the same origin.

**Figure 23:**
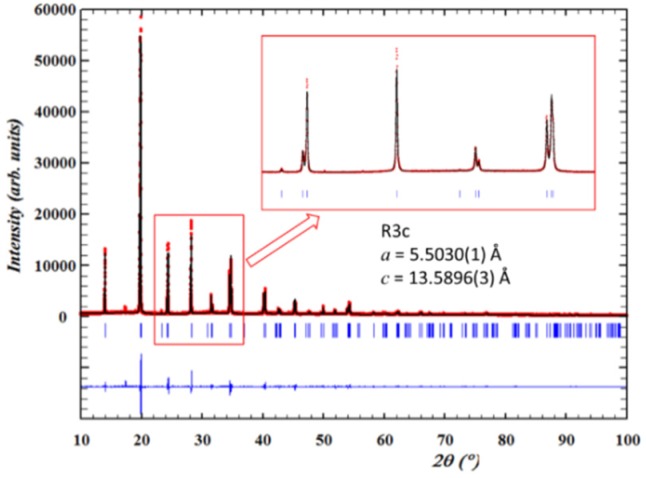
Synchrotron X-ray diffraction pattern and Rietveld analysis of powder flat specimen obtained by crushing the poled BNBT6 ceramic. Reliability factors: *R*p = 6.22, *R*wp = 8.61. Inset shows some relevant peaks of the rhombohedral *R*3*c* symmetry of the powder structure.

Figure 23 shows the experimental pattern and the Rietveld plots obtained for the powder after crushing the poled ceramic. As expected, the preferential orientation corresponding to a poled ceramic is lost. The figure inset reveals that the pattern no longer shows humps at the low angle side of the main perovskite peaks. Therefore, this result, though initially it may be a surprise, is not completely unexpected. It is also noticeable that the lattice parameters of the rhombohedral structure changes very little from the ceramic to the crushed sample.

It seems clear from this result that the experimental condition of random crystallite orientations virtually eliminates the structural contribution to the diffraction pattern that causes the humps. This suggests that such structural contribution is a highly directional one related with the experimental conditions of the measurement. In our poled ceramic experiment, only interplanar distances that are parallel to the electric field are detected. This means that humps are associated to crystallites whose morphology is nanosized, specifically in the direction of the electric field.

Humps could be the signature of planar defects such as those observed in BNBT5 [130] or be assigned to a near surface layer considered for relaxor ferroelectrics to accommodate the lattice distortion near the surface [131,132,133].

In our three-component model for the studied non-poled (ergodic relaxor) [87] and poled (ferroelectric) BNBT6 ceramics (present report), our plausible explanation of the experimental results is that humps reflect the lamellar crystallites of tetragonal (*P*4*mm*) symmetry observed by TEM in poled BNBT6 specimens [110]. The transition from the *P*4*bm* to the *P*4*mm* tetragonal symmetries was observed by TEM at the first poling stages of BNBT6 [110]. These lamellar *P*4*mm* crystallites could appear in the unpoled ceramic as a result of the intergranular stress arising during sintering of the synthetic powder (mechanical poling). They are un-affected by the field at the explored near-electrode surface of the poled ceramic in our measuring geometry. These lamellar crystallites would have a few tenths of nanometer size in the direction of the electric field, and their large faces would be oriented parallel to the sample surface. A negligible volume fraction of near-electrode surface, highly oriented, lamellar tetragonal phase that most probably cause the humps of the diffraction pattern was observed in our experiments on BNBT6. At the same time, we can unambiguously discard through this study the coexistence of rhombohedral and tetragonal ferroelectric global phases in BNBT6.

As a result of this study, we found a virtually single-phase, ferroelectric global rhombohedral (*R*3*c*) phase in BNBT6. The results of our Rietveld refinement are included in Table 4 and Table 5, below.

The structural characterization given to the considered ceramic is very much related with the piezoelectric properties of this material. The absence of coexisting global ferroelectric phases for BNBT6 composition prevents an enhanced remnant polarization in the material and the enhanced electromechanical properties, by similarity with what happens with the lead titanate-zirconate one [2], expected but not found for this composition of the MPB of the BNT-BT system.

### 4.3. XAFS

It has been well established that the MPB in the BNBT phase diagram represents the most worthy domain for the search of competitive ferro-piezoelectric properties. To our knowledge, the first Ti K-edge XANES study of this kind of materials was presented in the work [87]. The general conclusion in that work was that the phases present in the material are a globally pseudocubic *Pm-*3*m* perovskite and an averaged ferroelectric rhombohedral *R3c* phase. In [87] the comparison of the pre-edge peak type B of BNBT6 suggests that even in the unpoled state, BNBT6 samples, both powder and ceramic, have spontaneous polarization arising from the off-displacement of the Ti^4+^ ion from the center of the oxygen octahedra.

**Figure 24:**
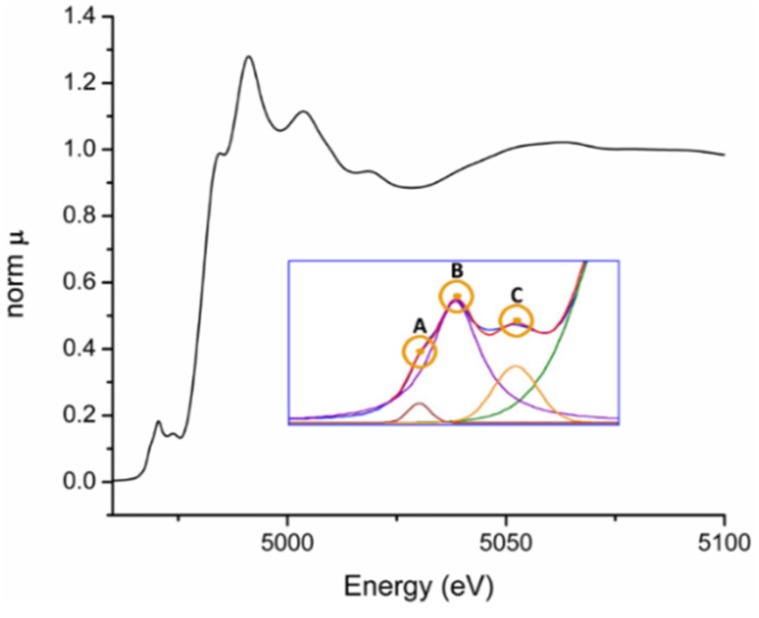
Normalized XANES spectrum of the Ti K edge of a BNBT4 powder sample, recorded at Stanford Synchrotron Radiation Lightsource. The inset corresponds to A, B and C modeled Lorentzian peaks of the pre-edge of XANES zone.

The general behavior of absorption coefficient from the lead-free material BNBT4 is presented in Figure 24. The XANES spectrum corresponds to the powder sample, prepared before pressing. In the same figure appears the modeling of the A, B and C features for the BNBT4 powder. Fitting was performed with Lorentzian shape for the B peak and the area was adjusted until satisfactory fitting was obtained.

Table 3 shows the values of the pre-edge peak B areas and uncertainties, expressed as standard deviations, from the reference compounds, a PbTiO_3_ thin film, BNBT4-powder and ceramic and the BNBT6-powder and ceramic presented in [87]. The same table presents an assessment of the proportionality constant K of Expression (2) for the calculation of the root mean square displacement 〈δ^2^〉^1/2^ of Ti atom from the centro-symmetric position. This evaluation was done by applying Expression (2) to the modeled area of pre-edge B features 〈*I_B_*〉 of the PbTiO_3_ thin film and the average Ti displacement reported in [134] and to the 〈*I_B_*〉 corresponding to the Ti displacement resulting of the structure presented in Table 4 below. This value K may be useful for evaluating the displacement of Ti from centrosymmetry in other experiments.

**Table 3:** XANES results for BNBT100*x*.

Sample	〈*I_B_*〉 (eV)	〈δ^2^〉 (A^2^)	K (eV A^3.5^)
PbTiO_3_	1.0 (2)	0.308 (4) ^a^	6.5 (1)·10^4^
BNBT4-powder	0.95 (9)	–	–
BNBT4-ceramic	0.86 (8)	–	–
BNBT6-powder	1.02 (9)	0.069 (6) ^b^	1.1 (2)·10^6^
BNBT6-ceramic	0.8 (1)	–	–

^a^ reference [134]; ^b^ diffraction component of present work. Uncertainties expressed as standard deviations.

Therefore, the table shows that BNBT4 and BNBT6 samples have, within uncertainties, practically the same areas and they are equal to that of the PbTiO_3_ thin film. These results show that all BNBT samples have practically equal Ti^4+^ cation displacement from symmetry center and confirm that even in the unpoled state, the BNBT samples—powder and ceramic—have spontaneous polarization arising from the off-displacement of the Ti^4+^ ion from the center of the oxygen octahedra. It should be taken into account, on one hand, that XANES depends of interatomic distances, and on the other hand, that our measurement has been performed on polycrystalline samples. Then, the pre-edge feature does not show easily if the displacement is toward the vertex or the face of the octahedron, especially in the case of a small translation. The different order of magnitude of the K value presented in Table 2 has its origin, probably, in experimental differences. The XAFS signal of the thin film should be less intense than that of the powder, and nevertheless, it produces an area 〈*I_B_*〉 comparable to that of BNBT samples.

### 4.4. BNBT MPB Summary

Integrating bibliographic review, diffraction and XAFS experiments, the characterization we propose for the BNBT morphotropic phase boundary is as follows.

Ceramic XRD patterns occasionally show humps caused by nanometric thickness lamellae (NTL), surface artifacts of tetragonal symmetry, lamellar morphology and nanometric thickness, oriented parallel to the sample surface.

- BNBT4 is essentially *R*3*c* + NTL;
- Unpoled BNBT6 contains *R*3*c* and pseudocubic global phase + NTL;
- Unpoled BNBT7 contains pseudocubic global phase + NTL;
- So-called pseudocubic phase consists of *P*4*bm* 3D nanodomains embedded in a cubic matrix;
- Under poling, BNBT6 shows *R*3*c* symmetry and BNBT7 becomes predominantly *P*4*mm*;
- Crystallographic data of considered representative phases are summarized in Table 4 and Table 5.

**Table 4:** BNBT*x* (*x* = 4, 6, 7) space groups and lattice parameters.

Material			Structure/Symmetry	*a* (Å)	*c* (Å)	Source
BNBT4 unpoled			*R*3*c* + NTL	5.4966(2)	13.5290(6)	[70]
BNBT6	Unpoled		*R*3*c*	5.505(1)	13.598(1)	[87]
			*P*4*bm* relaxor *+* NTL	≅5.5172	≅3.9010	
	Poled	Ceramic	*R*3*c* + NTL	5.5035(1)	13.5901(3)	Present report
		Milled	*R*3*c*	5.5030(1)	13.5896(3)	
BNBT7 unpoled			*P*4*bm* relaxor + NTL	5.5229(1)	3.9063(1)	[111]

**Table 5:** BNBT*x* (*x* = 4, 6, 7) atomic coordinates. To facilitate comparison with other reports, all the atomic coordinates have been referred to the most commonly used coordinate system.

Material	Atom	Wyck	*x*	*y*	*z*	SOF	ITF (B)	Source
BNBT4 *R*3*c* unpoled	Bi, Na, Ba	6a	0	0	0.250	0.48	Anisotropic factors given in [70]	[70]
	Ti	6a	0	0	−0.0043(1)	0.04		
	O	18b	0.1307(1)	0.3404(1)	0.0843(1)	1		
BNBT6 *R*3*c* poled, milled	Bi, Na, Ba	6a	0	0	0.250	0.47	2.88(3)	Present report
	Ti	6a	0	0	−0.0050(4)	0.06	1.92(6)	
	O	18b	0.146(1)	0.355(1)	0.0593(4)	1	2.7(2)	
BNBT7 *P*4*bm* unpoled	Bi, Na, Ba	2b	0	0.5	0.553(2)	0.465	Anisotropic factors given in [111]	[111]
	Ti	2a	0	0	0	0.07		
	O	2a	0	0	0.512(1)	1		
	O	4c	0.263(1)	0.237(1)	0.030(1)	1		

## 5. General Summary

Summary of considered synchrotron analysis techniques.

**Techniques****Revealed structural features**Diffraction/scattering1D *I* = *I*(2θ)High-resolution powder 1D-XRD; Rietveld analysis; Total scattering; Powder 1D with *in situ* variations of temperature and electric fieldQualitative and quantitative phase analysis; Global crystal structure (lattice parameters, space group, atomic positions); Microstructure (crystal and domain sizes, strain condition, texture); Electric-field induced phase transformations; Radial distribution function2D *I* = *I*(2θ, α)Debye ring analysis; Diffuse scattering; Single and polycrystal 2D reciprocal space investigations with *in situ* variations of temperature and electric fieldTexture; Chemical and structural local order-disorder phenomena; Crystallite and ferroelectric domains size and shape; Orientation dependence of the effect of electric field on structure, microstructure, polarization and strain conditionsXAFSXANESPre-edge peak intensities; Main edge transitions intensitiesRandomly oriented local polarization; Phase transitions; Lattice strain; Oxidation state; Density of states, MagnetizationEXAFSNearest neighbor distances (elemental specific); Coordination numbersRadial distribution function around absorbing atom
